# *Clostridioides difficile* Infection: Diagnosis and Treatment Challenges

**DOI:** 10.3390/pathogens13020118

**Published:** 2024-01-27

**Authors:** John E. Markantonis, John T. Fallon, Rajat Madan, Md Zahidul Alam

**Affiliations:** 1 Department of Pathology and Laboratory Medicine, Brody School of Medicine, East Carolina University, 600 Moye Boulevard, Greenville, NC 27834, USA; markantonisj22@ecu.edu (J.E.M.); fallonj19@ecu.edu (J.T.F.); 2 Division of Infectious Diseases, Department of Internal Medicine, University of Cincinnati College of Medicine, Cincinnati, OH 45267, USA; madanrt@ucmail.uc.edu; 3 Veterans Affairs Medical Center, Cincinnati, OH 45220, USA

**Keywords:** *Clostridioides difficile* infection, drug-resistant pathogen, nucleic acid amplification testing, host immunity, enzyme immunoassays, recurrent *C. difficile* infection, cell cytotoxicity neutralization assay

## Abstract

*Clostridioides difficile* is the most important cause of healthcare-associated diarrhea in the United States. The high incidence and recurrence rates of *C. difficile* infection (CDI), associated with high morbidity and mortality, pose a public health challenge. Although antibiotics targeting *C. difficile* bacteria are the first treatment choice, antibiotics also disrupt the indigenous gut flora and, therefore, create an environment that is favorable for recurrent CDI. The challenge of treating CDI is further exacerbated by the rise of antibiotic-resistant strains of *C. difficile*, placing it among the top five most urgent antibiotic resistance threats in the USA. The evolution of antibiotic resistance in *C. difficile* involves the acquisition of new resistance mechanisms, which can be shared among various bacterial species and different *C. difficile* strains within clinical and community settings. This review provides a summary of commonly used diagnostic tests and antibiotic treatment strategies for CDI. In addition, it discusses antibiotic treatment and its resistance mechanisms. This review aims to enhance our current understanding and pinpoint knowledge gaps in antimicrobial resistance mechanisms in *C. difficile*, with an emphasis on CDI therapies.

## 1. Introduction 

With an estimated incidence rate of 110.2 cases per 100,000, *C. difficile* infection (CDI) is one of the leading causes of morbidity and mortality due to infectious diarrhea in the United States [1]. CDI occurs when there is a shift in the colonic microbial flora allowing toxin-producing strains of the Gram-positive, spore-forming, anaerobic bacillus to over proliferate [2]. Antibiotic exposure, the most important risk factor for CDI, results in a reduction in the population of non-pathogenic anaerobes that normally inhabit the gut [2,3]. This leads to a decrease in competition for space and resources for *C. difficile* allowing it to replicate unchecked [2]. Additionally, an ineffective host immune response contributes to this disease process due to the reduced elimination of this pathogenic bacterium as well as an increased inflammatory response to the bacterium and its toxins [2,4,5,6,7]. Clinical manifestations of CDI commonly include fever, leukocytosis, abdominal pain and profuse watery diarrhea [8,9]. Severe complications from CDI include dehydration, electrolyte imbalances, acute kidney injury (AKI) and pseudomembranous colitis [8,9,10]. The presence of toxic megacolon, ileus or shock indicates fulminant (severely complicated) disease which requires aggressive medical therapy [8,9,10].

The *C. difficile* bacterium produces clostridial toxins, which are its major virulence factors, and are responsible for CDI [2,11]. CDI generally occurs from strains that produce two exotoxins, toxin A (*tcdA*) and toxin B (*tcdB*) [2,11,12]. Rare toxigenic strains that harbor mutations in *tcdA* have been reported throughout the world [13,14,15]. These strains lack toxin A production; however, they still retain the ability to produce toxin B [13,14]. Interestingly, these toxin B-only producing *C. difficile* strains are still strongly associated with CDI [13,14]. In contrast, strains that produce only toxin A, as well as non-toxigenic strains, are rarely associated with pathogenicity [11,13,14,16]. A third toxin (clostridium binary toxin, CDT) has been identified in approximately 20% of *C. difficile* strains [2,11,12,17,18]. Strains that produce CDT, such as PCR ribotype 027/North American pulse-field type 1, restriction endonuclease analysis type B1 strain (NAP1/B1/027 or RT-027), are often associated with severe disease and are known as hypervirulent strains [11,15,17,18]. In addition to CDT production, mutations in the toxin regulator gene (*tcdC*) have been found in these strains, possibly leading to hyperproduction of toxin A and toxin B [11,18,19,20]. The NAP1/B1/027 strain is notable not only for its heightened toxin production but also for an increased sporulation rate, potentially enhancing the pathogen’s survival and promoting the spread of CDI [21].

*C. difficile* was discovered in 1935 and was successfully isolated from the stool of healthy infants [11]. It was initially considered part of the normal flora of the human gastrointestinal tract, and commonly, that is the case [11]. In the 1970s, its association with antibiotic-associated diarrhea and its nature as a pathogen was first realized [11]. During the early 2000s, the hypervirulent *C. difficile* strain RT-027 acquired fluoroquinolone-resistance and several epidemic outbreaks have occurred in North America and Europe periodically since that time [19,22,23]. Prevalent strains in the US currently include ribotypes (RT) 027, 106, 014/20 and 002 [15]. In addition to RT-027, hypervirulent strains RT-078 and RT-244 are also present, but with a much lower prevalence rate [15]. Of note, RT-017 has emerged as the major cause of CDI outbreaks in Asia and Africa [15,24]. Distinct *C. difficile* strains continue to emerge globally making CDI a worldwide issue [15].

## 2. Laboratory Tests to Diagnose CDI

### 2.1. Toxigenic Culture

Culturing viable organisms from stool followed by the confirmation of toxin production is considered the “gold standard” for diagnosing CDI [25]. Cycloserine–cefoxitin–fructose–egg yolk agar (CCFA), or a modified version, is the standard media used for the isolation of *C. difficile* [9,25,26]. Fresh stool samples should be treated with alcohol or heat shock to facilitate the conversion of spores to their vegetative forms prior to inoculation on CCFA or a similar selective media [9,26]. This is followed by anaerobic incubation at 37 °C for 48 h or longer [9,25,26]. Colonies with the typical appearance of *C. difficile* (flat, yellow, ground-glass-appearing colonies with a yellow halo) are selected for Gram staining and confirmatory testing [26,27]. This is generally accomplished by either biochemical analysis or through matrix-assisted laser desorption ionization time-of-flight mass spectrometry (MALDI-TOF) [9,25]. Differentiating *C. difficile* from other *Clostridia* can be accomplished by identifying motility, gelatin hydrolysis, glucose fermentation and esculin hydrolysis, while demonstrating the negative production of lecithinase, lipase, indole and urease [28]. Chromogenic media are commercially available, allowing for direct plating and a reduced incubation time (24 h), but are generally a more costly alternative to standard selective media [25]. Confirmed isolates are then tested for toxin production using a cell cytotoxicity neutralization assay (CCNA) [9,25,26,27]. Although considered the “gold standard”, the long turn-around-time and high complexity of this testing makes its routine use in clinical labs impractical [9]. Its current use is largely confined to research labs. 

### 2.2. Cell Cytotoxicity Neutralization Assay

In addition to testing for toxin production from cultured *C. difficile* isolates, CCNA can be performed directly on fresh stool which allows for the detection of in vivo toxin production. For this testing, a stool filtrate is generated followed by the application of the supernatant to a cell line monolayer, commonly a human foreskin fibroblast-derived line [9,27,29]. Additionally, a second cell culture is incubated with a toxin-specific antibody following the application of the stool filtrate supernatant [9,27,29]. The monolayers are examined under high power microscopy at designated times over 48 h for signs of cytopathic effects (CPE) [9,27,29]. CPE refers to observable cellular changes, particularly cell rounding, resulting from the inactivation of Rho proteins. *C. difficile* toxins induce CPE by glucosylating low-molecular-weight GTP-binding proteins from the Rho subfamily, leading to actin reorganization and cell rounding [30]. This cell culture-based test is interpreted as being positive if ≥50% of the cultures cells exhibit cell rounding (CPE) with no CPE identified in the cells from the antibody treated culture. When performed properly this testing has high clinical sensitivity and specificity; however, several factors limit its clinical utility [9]. It has a prolonged turn-around-time and subjective interpretation issues compared to antigen- and molecular-based testing [9]. Additionally, as most clinical microbiology labs have transitioned to molecular testing for most assays, maintaining cell lines for this testing is often not practical. Like toxigenic culture, this testing is best used for reference testing in a research setting [9].

### 2.3. Nucleic Acid Amplification Testing

Molecular testing allows for the rapid and analytically sensitive detection of toxigenic *C. difficile* from clinical samples. Examples of nucleic acid amplification testing (NAAT) methods routinely used in clinical microbiology labs include polymerase chain reaction and loop-mediated isothermal amplification [9,31,32,33]. NAAT detects genes specific to toxigenic *C. difficile*, most commonly toxin-related genes [34]. Most molecular assays contain the toxin B gene target (*tcdB*); however, some assays also contain a target for *tcdA*, *cdt* and/or *tcdC* [34]. Limitations of NAAT include a higher average cost than alternative testing for the detection of asymptomatic colonization [9]. Among healthy adults, *C. difficile* colonization without clinical signs of CDI ranges from 0% to 17.5%, while hospitalized adults show a higher prevalence, ranging from 0% to 51% [35,36]. Molecular testing does not detect active toxin production, thus leading to specificity issues in the diagnosis of CDI when used alone [9]. Unnecessary treatment of asymptomatic colonized individuals can lead to adverse effects [35].

### 2.4. Enzyme Immunoassays

Antigen testing, most commonly in the form of enzyme immunoassays (EIAs), provides a rapid, simple and cost-effective alternative to other diagnostic tests for CDI. This form of testing had previously fallen out of favor due to poor analytical performance; however, newer technological advancements have significantly improved these methods’ clinical performance [37]. EIAs utilize antibodies that bind specifically to the antigen of interest [38]. EIAs that target toxin A and toxin B (toxin EIA) are considered the most specific diagnostic testing method routinely used by clinical microbiology labs for CDI diagnosis [8,9,39,40]. Since clostridial toxins degrade rapidly, the toxin EIA testing has relatively poor sensitivity [41,42]. This necessitates the use of a second, more sensitive, test alongside toxin EIA testing [9,41,43]. Glutamate dehydrogenase (GDH) is an antigen found in high levels in *C. difficile* and is not present in appreciable levels in other related organisms [44]. This creates an EIA that detects GDH (GDH EIA) a suitable screening target for the presence of *C. difficile*. The presence of GDH does not distinguish between toxigenic *C. difficile* and non-toxigenic strains [44]. This can be problematic as a positive result may indicate colonization as opposed to CDI, especially in individuals with a low test probability for CDI [44]. Thus, the detection of CDI by EIA is best optimized by pairing GDH and toxin detection [9,41,44]. 

### 2.5. Current Landscape of Clinical CDI Testing

Like syphilis testing, no single stand-alone test is currently recommended for the optimal clinical diagnosis of CDI [9,45,46]. An algorithmic approach is best suited for this task [8,9,41,46,47,48]. Current recommendations include a sensitive test (GDH EIA, NAAT) as the first step in CDI screening followed by a specific test (toxin EIA) to confirm in vivo toxin production [8,9]. For institutions that utilize a GDH EIA as the first part of the algorithm, a *C. difficile* NAAT can be used to arbitrate specimens that are GDH+/Toxin− to confirm whether the detected organism is a toxigenic strain [8,9]. This can be carried out reflexively or in all patients in whom the pre-test probability for CDI is high. Toxigenic culture and direct stool CCNA offer the best sensitivity/specificity but are practically illogical for most clinical laboratories [9]. Ultimately, there is no one test or algorithm that has a strong literature to support its use over others [49]. However, it is important to note that depending solely on molecular tests may result in overdiagnosis, unnecessary treatment and elevated healthcare costs associated with CDI. A study conducted by Polage at al. aimed to determine the natural history and treatment necessity for patients testing Tox−/PCR+ (toxin immunoassay negative, polymerase chain reaction positive) for CDI [50]. Among 1416 hospitalized adults, 21% were PCR positive, but only 44.7% of these had toxins that were detected by clinical tests. Tox−/PCR+ patients showed a lower bacterial load, less antibiotic exposure and fewer complications compared to Tox+/PCR+ patients. The median duration of diarrhea was shorter in Tox−/PCR+ patients, and no CDI-related complications or deaths occurred, unlike in Tox+/PCR+ patients. The study suggests that relying solely on molecular tests for CDI diagnosis may lead to overdiagnosis, overtreatment and increased healthcare costs. The decision for which testing should be used for the diagnosis of CDI is largely decided by the laboratory in conjunction with their associated clinical staff leadership. It is important to note that, while laboratory testing is supportive for diagnosis, CDI should not be eliminated from the differential diagnosis for individuals with significant risk factors for and a clinical presentation consistent with CDI based solely on laboratory results [8,9]. 

## 3. Treatment of *C. difficile* Infection 

### 3.1. Treatment of the Initial Episode of CDI

The treatment of CDI, which was initially considered relatively straight forward, has become more challenging as antibiotic resistant and hypervirulent strains have emerged [19,51,52,53]. The current standard of care (SOC) for CDI treatment is largely based on recently published recommendation guidelines from the Infectious Disease Society of America/Society for Healthcare Epidemiology of America (IDSA/SHEA), American College of Gastroenterology (ACG) and the European Society of Clinical Microbiology and Infectious Disease (ESCMID) [8,9,10,54]. However, it should be noted these recommendations are based on low levels of evidence and lack high-quality research evidence [49]. 

The management of CDI primarily relies on three antibiotics: metronidazole, vancomycin and fidaxomicin, which are routinely employed in its treatment [8,10,54]. Metronidazole belongs to the nitroimidazole class of drug that is highly effective in the treatment of anaerobic bacterial infections and certain parasites [55]. Its mechanism of action is based on generation of reactive free radicals that damage nucleic acid. For the treatment of CDI, it can be administered orally or intravenously [8,10,54]. Vancomycin, a glycopeptide, prevents crosslinking of D-Ala-D-Ala moieties in peptidoglycan leading to impairment in cell wall synthesis and stability [56]. It has activity predominantly towards Gram-positive bacteria [56]. Vancomycin is minimally absorbed by the intact gastrointestinal tract and concentrates at high levels in the colon lumen, the site of its intended antimicrobial effect [57]. Although relatively new for CDI treatment, fidaxomicin has established an important role in the treatment of this disease [8,10,54,58]. Belonging to the macrocyclic lactones (macrolide) class of antimicrobial agents, fidaxomicin is unique in its narrow spectrum of anti-bacterial activity [58]. It effectively targets *C. difficile* without disrupting much of the remaining colonic flora [58,59]. Research studies have shown a decreased rate of treatment failure and recurrence compared to treatment with metronidazole or vancomycin [60,61,62]. In addition to initiating *C. difficile*-targeted antimicrobial therapy discontinuing non-CDI antimicrobials, if being administered, allows for the re-establishment of the normal colonic flora [8,10,54]. Fluid resuscitation is also important in CDI treatment to prevent complications associated with dehydration [8,10,54].

Standard-of-care (SOC) treatment for an initial episode of CDI involves a treatment course based on either fidaxomicin or oral vancomycin [8,10,54]. Per IDSA guidelines, fidaxomicin and vancomycin are recommended as the SOC for adults, while vancomycin and metronidazole continue to be considered standard for pediatric patients [9,54]. Fidaxomicin (200 mg) taken orally twice a day for 10 days is the preferred first-line treatment in these cases as its narrow spectrum likely leads to less gut dysbiosis and lower *C. difficile* recurrence rates [8,10,54]. ESCMID guidelines recommends the consideration of an extended course of fidaxomicin (200 mg twice daily for 5 days, then 200 mg every other day for 7–25 days) for patients at high risk for recurrence (e.g., geriatric patients, continued use of antibiotics and/or proton pump inhibitors, etc.) [10]. Fidaxomicin is significantly more expensive than oral vancomycin and not available at all treatment facilities [8,10,54]. An acceptable alternative is 125 mg vancomycin taken orally fourtimes a day for ten days [8,10,54]. If both agents are unavailable, 500 mg metronidazole taken by mouth three times a day for 10–14 days can be considered in initial, non-severe, cases [8,10,54]. Per ACG guidelines, metronidazole can also be considered over fidaxomicin and oral vancomycin for use in initial episodes of non-severe CDI in low-risk patients [8]. Severe cases are generally defined by a high fever, marked leukocytosis and the development of acute kidney injury [8,10,54].

For fulminant (severe complicated) CDI, treatment is the same regardless of whether it is an initial episode or a recurrence [8,10,54]. Fulminant CDI is largely defined as the development of profound hypotension/shock, toxic megacolon, ileus or other signs of rapid deterioration in medical condition [8,10,54]. IDSA/SHEA and ACG guidelines suggest the administration of 500 mg vancomycin by mouth or nasogastric tube every 6 h combined with 500 mg metronidazole administered intravenously every 8 h [8,54]. If ileus is present, rectal vancomycin administration (500 mg every 6 h) should be considered [8,54]. ESCMID guidelines differ from IDSA/SHEA and ACG, in that there is no recommendation to increase the dose or frequency of the administration of vancomycin [10]. The guidelines cite concerns for increased adverse effects and the development of antimicrobial resistance [10]. The basis of this recommendation is that, as the standard dose already results in high colonic intraluminal concentrations, the therapeutic benefits of the higher dose are uncertain [10]. The ESCMID guidelines also state the adjunctive additions of intravenous metronidazole and/or intravenous tigecycline for individual’s with a deteriorating SOC and CDI antimicrobial agents can be considered on a case-by-case basis; however, their routine use is not recommended [10]. Early surgical consultation is recommended for severe and fulminant cases of CDI as prompt surgical intervention when indicated may lead to less aggressive surgical procedures and better surgical outcomes [8,9,10].

### 3.2. Treatment of Recurrent CDI

Recurrent CDI (rCDI) is generally defined as the return of symptoms consistent with CDI within 8 weeks of an initial episode with laboratory confirmation [8,9,10,63]. For rCDI episodes, anti-*C. difficile* antimicrobial agents remain the backbone of medical therapy [8,10,54]. Novel treatment strategies incorporating toxin-binding monoclonal antibodies and fecal microbiota transplantations have now become established in treatment courses for rCDI cases [8,10,54,64]. For a first recurrence of CDI, fidaxomicin remains the preferred treatment option per most societal guidelines [8,10,54]. The standard 200 mg dose can be given twice a day for 10 days, or an extended course where the standard dose is given twice a day for 5 days followed every other day for 20 days based on IDSA/SHEA guidelines or 7–25 days if following ESCMID guidelines [10,54]. Alternatively, oral vancomycin in a standard 10-day course or in a tapered/pulsed-dosed regiment can be considered [8,10,54]. The ACG guidelines strongly recommend the use of fidaxomicin if oral vancomycin or metronidazole was the treatment agent used in the initial CDI episode and tapered/pulsed dosing of oral vancomycin over a standard course when used in recurrence [8].

For second and subsequent recurrences, the IDSA/SHEA guidelines recommend either a standard or extended course of fidaxomicin, a tapered/pulsed-dose oral vancomycin regiment or a standard course of oral vancomycin followed by rifaximin 400 mg three times daily for 20 days [54]. The ACG and ESCMID guidelines recommend fecal microbiota transplantation (FMT) as the first-line treatment option for second and subsequent recurrences [8,10]. FMT has been shown to be effective in preventing recurrence in individuals who have failed SOC antimicrobials in the past [65,66,67,68,69]. The goal of FMT is to restore a functioning gut microbiome to suppress the growth of *C. difficile* by competing for resources and epithelial surface area [67]. If FMT is not a feasible option, SOC antimicrobials can be considered [10]. In the FMT procedure, stool samples from healthy donors are chosen for transplantation into the recipient’s colon. The preferred methods for this transplantation include ingestion through an oral capsule or administration via a colonoscopy [65,69]. It is important to note that a rectal enema is another option, although it is not recommended according to ACG guidelines [8,69]. The ACG and ESCMID guidelines state that FMT can also be considered for severe and fulminant CDI cases where individuals on SOC therapy are failing, and a surgical intervention is not feasible [8,70]. 

On 30 November 2022, the FDA announced the approval of Rebyota as a preventive measure for rCDI in individuals aged 18 and above who have undergone antibiotic treatment [71]. Rebyota is a rectally administered, pre-packaged, single-dose microbiota suspension of 150 mL. Its effectiveness has been evaluated through randomized, double-blind, placebo-controlled, multicenter studies, demonstrating that Rebyota is well-tolerated and safe for use in adults with rCDI [72,73]. Additionally, the FDA recently approved Vowst as the first orally administered fecal microbiota product for preventing CDI recurrence following antibacterial treatment [74]. Vowst, containing live bacteria, is derived from human fecal matter donated by qualified individuals, with a dosing regimen of four capsules taken orally once a day for three consecutive days [74].

While effective in managing rCDI, FMT poses a potential risk of transmitting infectious agents. IDSA/SHEA guidelines recommend reserving FMT for individuals with two prior recurrences based on the concern for adverse events. These include the inadvertent transplantation of antimicrobial-resistant or pathogenic organisms and the development of sepsis due to these newly introduced gut microorganisms. It should be noted that these are rare occurrences with this procedure [54,66,67,75,76]. Additionally, although FMT poses the risk of transmitting multi-drug resistant pathogens, the FDA’s approval ensures that these products meet certain safety and efficacy standards for clinical use, potentially reducing associated risks [77]. 

Bezlotoxumab is a monoclonal antibody that binds and neutralizes toxin A and toxin B [78]. Several studies have shown decreased recurrence rates when it is administered alongside SOC antimicrobial therapy for CDI [79,80]. This is especially evident in the case of oral vancomycin, as this was the antimicrobial agent largely used in these clinical studies [8,54,70]. Data on its use with fidaxomicin are limited [8,54,70]. Congestive heart failure (CHF) is also a relative contraindication for its use; its benefit in prevention of CDI recurrence needs to be weighed against the potential risk of CHF exacerbation [8,54,70]. Its incorporation into the treatment course as a one-time dose administered intravenously for both the first and subsequent recurrence is highly recommended [8,54,70]. It should also be considered in patients at high risk for recurrence even during an initial CDI episode [8,54,70]. Managing recurrent CDI poses a significant challenge, requiring attention in both treating the underlying infection and implementing preventive measures for future episodes in every treatment plan.

## 4. Antimicrobial Resistance in *C. difficile* and Its Mechanisms of Resistance

As mentioned earlier, the management of CDI primarily hinges on three antibiotics: metronidazole, vancomycin and fidaxomicin [8,54]. For over three decades, metronidazole and vancomycin have stood as the frontline treatments, while fidaxomicin, gaining approval in 2011, has predominantly been employed for managing recurrent CDI [49,54,61,70]. Despite their historical efficacy, there have been documented instances of *C. difficile* isolates demonstrating diminished susceptibility and, in some cases, resistance to these antibiotics [81,82,83]. Moreover, the use of various other antimicrobials, including ampicillin, cephalosporins, fluoroquinolones and clindamycin, has been identified as a risk factor for CDI and the emergence of epidemic strains of *C. difficile*, which are resistant to multiple antibiotics [84,85,86]. For example, the extensive utilization of fluoroquinolones in North America preceded the rise and dissemination of fluoroquinolone-resistant RT027 strains, catalyzing the global surge in CDI in the early 2000s [23,87,88]. Furthermore, measures such as curtailing the prescription of fluoroquinolones have been correlated with a reduction in infections attributed to fluoroquinolone-resistant *C. difficile* isolates, potentially elucidating the decline in CDI in the UK. 

After antibiotic treatment, over 20% of patients encounter rCDI, and among these individuals, 45–65% undergo multiple subsequent episodes [89,90]. The impact of antibiotic failure or resistance on treatment outcomes and the initiation of rCDI remains uncertain. Strikingly, the assessment of CDI treatment outcomes often overlooks antimicrobial resistance, given that anaerobic susceptibility testing of patient isolates is not routinely conducted in the diagnostic evaluation of CDI. Nevertheless, the growing instances of resistance to both conventional and newer CDI antibiotics, such as fidaxomicin, necessitate a reassessment of this perspective [15,49,91,92].

The rise and dissemination of antibiotic-resistant *C. difficile* isolates, particularly within the hypervirulent *C. difficile* ribotype 027 strains, poses a growing challenge in the treatment of CDI. This section will review the issue of antibiotic resistance and the mechanisms of resistance related to commonly used antimicrobial drugs for CDI management.

### 4.1. Metronidazole Resistance

For three decades, metronidazole was the recommended primary treatment for CDI [9]. However, recent evidence indicates that it has fewer clinical benefits compared to vancomycin [9]. Due to this decreasing effectiveness, current guidelines from the IDSA/SHEA and ESCMID no longer endorse metronidazole as the first-line treatment for adult CDI. This marks a significant change in how CDI is treated. Presently, metronidazole is recommended only for the initial episode of non-severe CDI in situations where access to vancomycin or fidaxomicin is limited [9]. Alternatively, metronidazole may be reserved for intravenous therapy in combination with vancomycin for severe CDI [9].

Metronidazole belongs to the bactericidal nitroimidazole class of antibiotics and is administered as a prodrug [55,93]. Within the cell, it undergoes activation through reactions facilitated by oxidoreductases, such as pyruvate-ferredoxin/flavodoxin oxidoreductase [55]. This activation results in the formation of reactive species that cause damage to nucleic acids and proteins while depleting cellular thiols. The reduction in its nitro group takes place via anaerobic enzymatic reactions with low redox potentials, resulting in cytotoxicity and the death of anaerobic bacteria [55,93,94]. The reductive activation process itself can be potentially cytotoxic, as metronidazole acts as an alternative electron acceptor, disrupting the proton motive force and inhibiting ATP production [55,93,95].

Over the past two decades, metronidazole has exhibited diminishing effectiveness compared to vancomycin. This trend was initially noted in a randomized clinical trial spanning from 1994 to 2002, where vancomycin achieved a cure rate of 97%, while metronidazole demonstrated a cure rate of 84% [96]. Subsequent clinical research conducted from 2005 to 2007 further underscored vancomycin’s superior cure rates compared to metronidazole, recording rates of 81.1% and 72.7%, respectively [97]. These studies emphasize a decline in the efficacy of metronidazole, particularly during the epidemic era. The decreased effectiveness of metronidazole in treating CDI is thought to have multiple contributing factors [98,99,100,101]. One potential explanation is that the heightened usage of metronidazole creates selection pressures, facilitating the emergence of drug-resistant strains of *C. difficile* [98,99].

**Figure 1:**
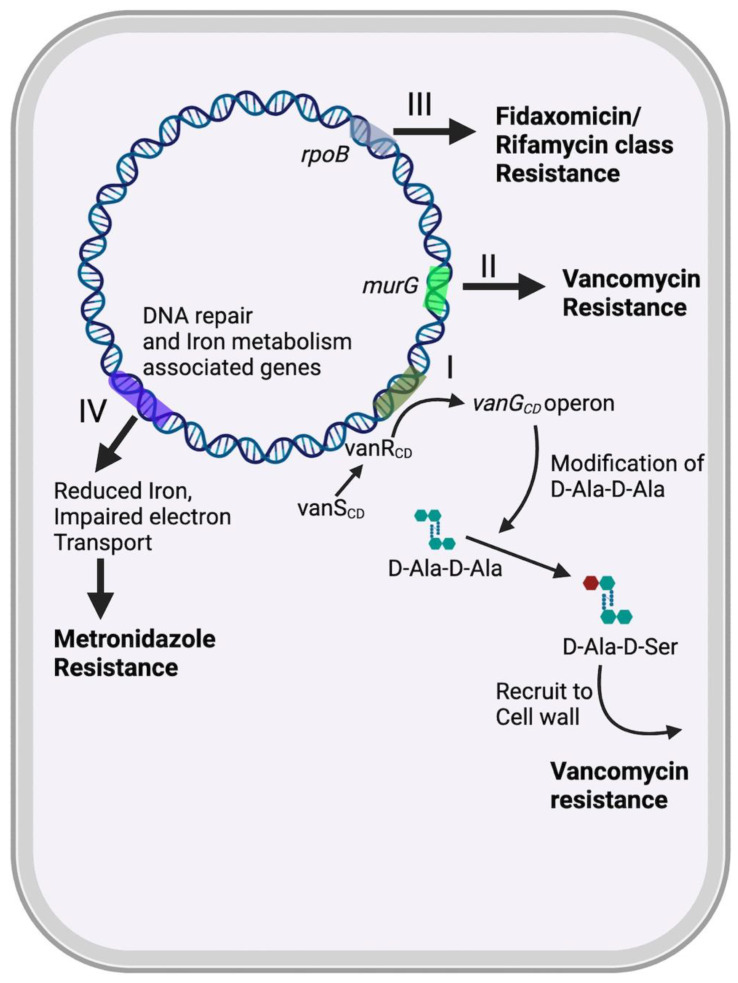
The diagram depicts the mechanisms by which *C. difficile* develops resistance to commonly utilized antibiotics in the treatment of CDI, encompassing vancomycin, metronidazole, fidaxomicin and rifamycins. (**I**) Vancomycin functions by tightly binding to the D-Ala-D-Ala C-terminus of uracil diphosphate-N-acetylmuramyl-pentapeptide, impeding the transglycosylation reaction responsible for incorporating late precursors into the developing peptidoglycan chain. This action inhibits the synthesis of the bacterial cell wall. Resistance to vancomycin in *C. difficile* is linked to mutations in the VanS_CD_ sensor histidine kinase and VanR_CD_ response regulator of the vanG operon-like gene cluster, *vanG_CD_*. These mutations modify peptidoglycan precursors, altering the vancomycin binding site and contributing to the emergence of vancomycin resistance in *C. difficile*. (**II**) An additional mechanism contributing to vancomycin resistance is associated with a point mutation in MurG N acetylglucosaminyltransferase. This mutation impacts the conversion of peptidoglycan precursor lipid I to lipid II, a crucial step in bacterial cell wall synthesis. (**III**) Fidaxomicin exerts its bactericidal effects by inhibiting bacterial RNA polymerase, thereby disrupting transcription and subsequent protein synthesis. Resistance to fidaxomicin in *C. difficile* has been linked to induced mutations in the RNA polymerase subunit-β (*rpoB*). In contrast, rifamycins hinder bacterial RNA synthesis by binding to the β subunit of RNA polymerase, RpoB, at a distinct site and step of RNA synthesis compared to fidaxomicin. Rifamycin resistance is associated with mutations in the rifamycin resistance-determining region of *rpoB*, identified in clinical isolates of *C. difficile*. (**IV**) Metronidazole induces DNA strand breakage and cytotoxicity, leading to bacterial cell death. Resistance in *C. difficile* to Metronidazole may arise through mechanisms that impede the formation of the active drug form, potentially mediated by multigenetic processes associated with oxidoreductive and iron-dependent metabolic pathways.

The resistance of *C. difficile* to metronidazole has been found to be associated with impaired intracellular iron content (Figure 1). In a study conducted by Deshpande et al., a laboratory-generated *C. difficile* mutant with a truncated feoB1 gene (encoding a ferrous iron transporter) exhibited reduced intracellular iron levels and a low level of resistance to metronidazole [102]. The authors suggested that a decrease in intracellular iron shifts cells toward flavodoxin-mediated oxidoreductase reactions, consequently hindering cellular effectiveness of metronidazole. Additionally, another study analyzing the metronidazole-resistant CD26A54_R isolate through proteomic analysis revealed a significant increase in the expression of the ferrous iron transport B (*FeoB*) protein in the absence of metronidazole [103]. This observation suggests that deficiencies in iron uptake and/or regulation may be linked to the development of metronidazole-resistant strains.

The Ferric Uptake Regulator (*Fur*) protein, a regulatory protein that governs the transcription of various genes in response to iron availability and oxidative stress, has been linked to metronidazole resistance [104]. Genomic analysis of serially passaged metronidazole-resistant CD26A54_R strain identified a point mutation (Glu41Lys) in the *fur* gene, which is absent in metronidazole-susceptible variant CD26A54_S of this strain [105]. Nevertheless, the precise role of this mutation in metronidazole resistance in *C. difficile* remains unclear.

Proteins engaged in electron transfer reactions play a pivotal role in the reduction of metronidazole, leading to the activation of the drug [55,106]. Analyses of *C. difficile*’s clinical isolate CD26A54_R, which sustained resistance to metronidazole through serial passages at sublethal concentrations, revealed mutations in genes linked to electron transport [105]. More precisely, the gene *glyC*, encoding glycerol-3-phosphate dehydrogenase, displayed an Ala229Thr mutation, while the gene *nifJ*, encoding pyruvate-flavodoxin oxidoreductase (PFOR), exhibited a Gly423Glu mutation [105]. Another in vitro study underscored the importance of PFOR in the context of metronidazole resistance in *C. difficile* [102].

Specific mutations found only in the metronidazole-resistant variant CD26A54_R, which underwent serial passages, indicate a possible connection to nutrient limitation and the abnormal growth observed in its culture [105]. Notably, the frameshift mutation Tyr214fs in the *hemN* gene, responsible for encoding oxygen-independent coproporphyrinogen III oxidase involved in hem biosynthesis, and a Ser328Phe mutation in the *thiH* gene, encoding a thiamine biosynthesis protein peptidase, are among the identified mutations [105]. These genetic alterations are proposed to contribute to nutrient scarcity, potentially influencing the aberrant growth characteristics observed in the strain’s culture.

Interestingly, heme plays a crucial role in accurately identifying metronidazole resistance in *C. difficile*, with most metronidazole-resistant strains demonstrating heme-dependent resistance [107]. A recent study revealed that epidemic strains underwent a shared mutation in the regulatory promoter of 5-nitroimidazole reductase (CDR20291_1308, annotated as *nimB*), transforming it from a latent to a consistently expressed resistance gene [108]. Additionally, the study demonstrated that the protein *C. difficile* NimB (*CdNimB*) functions as a heme-binding flavoenzyme, biochemically deactivating 5-nitroimidazoles to corresponding amines, with a substrate profile extending to 4-nitrobenzoic acid and 2-nitroimidazole. This study emphasizes the significance of heme in the context of metronidazole resistance, as it is intricately involved in regulating expression of key resistance genes like *nimB*.

These findings collectively shed light on the diverse mechanisms shaping metronidazole resistance in *C. difficile*, emphasizing the need for further exploration to comprehend the intricate interplay of genetic and environmental factors in resistance evolution.

### 4.2. Vancomycin Resistance

Vancomycin, initially considered a last-resort drug for severe infections, is now recommended as the first-line therapy for initial, recurrent and fulminant CDI [9]. Although this glycopeptide antibiotic was found to be superior in treating CDI compared to metronidazole, recent years have seen emerging strains with resistance or reduced susceptibility to vancomycin, raising significant concerns [109,110,111]. Notably, there has been an increase in strains with reduced susceptibility, as indicated by a rise in the minimum inhibitory concentration (MIC90) from 1 μg/mL for isolates from 1984 to 2003 to 4 μg/mL for isolates from 2011 to 2012 [110]. This shift suggests a growing challenge in treating CDI with vancomycin.

Initially hailed as a powerful antimicrobial with resistance-immune properties, vancomycin encountered challenges when reports of vancomycin-resistant *Enterococcus* species emerged in 1988, succeeded by *Staphylococcus aureus* in 2002 [56,112]. Vancomycin resistance mechanisms in enterococci are well documented, involving the modification of the terminal D-Ala with either D-Lac or D-Ser [56,113]. The *vanA* and *vanB* gene clusters encode high-level resistance, characterized by D-Ala-D-Lac, whereas low-level resistance is attributed to D-Ala-D-Ser, encoded by the *vanC*, *vanE* and *vanG* gene clusters [112,113]. *vanG*, an inducible chromosomal operon, induces vancomycin resistance in Enterococci through a sensor operon and a resistance operon. These work in tandem to produce the altered peptidoglycan precursor D-Ala-D-Ser. The sensor operon comprises a two-component regulatory system with a membrane-bound sensor histidine kinase (vanS) and a response regulator (vanR) transcriptional activator [56,113,114]. Upon exposure to vancomycin, this system activates the expression of subsequent resistance genes.

A gene cluster resembling the *vanG* operon, named *vanG_CD_*, has been identified in approximately 85% of clinical isolates of *C. difficile* [109,111]. Historically, the functional presence of this gene cluster did not demonstrate a direct role in mediating vancomycin resistance in *C. difficile*. However, recent findings indicate that mutations within the genes of this cluster are associated with vancomycin resistance in newly identified strains exhibiting unique genomic sequences and antibiotic resistance patterns (Figure 1). It has been observed that constitutive expression of *vanG_CD_* occurs in vancomycin-resistant clinical strains and laboratory-generated mutants, which harbor mutations in the vanSR two-component system that governs *vanG_CD_* [109].

Additional mechanisms, such as mutations in specific genes (Figure 1), have been proposed to account for vancomycin resistance in *C. difficile*. Genetic alterations were observed in certain strains and clinical isolates that were exposed to increasing vancomycin concentration, resulting in reduced susceptibility to the antibiotic [92]. One notable mutation, Pro108Leu, was identified in MurG N-acetylglucosaminyltransferase, responsible for catalyzing the conversion of peptidoglycan precursor lipid I to lipid II—an essential step in bacterial cell wall synthesis [92]. The identical strain displayed two extra mutations: a Glu327stop substitution in the presumed RNA/single-stranded DNA exonuclease CD3659 and removal of a solitary amino acid within a sequence of alanines in l-Ser deaminase encoded by sdaB gene [92]. This genetic change possibly mediates resistance by affecting multiple gene expression pathways.

Recently, a plasmid-mediated decrease in vancomycin susceptibility has been documented in isolates from patients unresponsive to vancomycin therapy [115]. The plasmid, named pX18–498, is a broad-host-range plasmid containing 51 ORFs, including a gene that encodes a putative N-acetylmuramoyl-L-alanine-amidase, a peptidoglycan remodeling enzyme. Introducing plasmid pX18–498 into a strain susceptible to vancomycin led to reduced susceptibility to the antibiotic [115]. The potential clinical importance of pX18–498 was illustrated by observing that mice infected with *C. difficile*-pX18–498 and treated with vancomycin displayed a higher *C. difficile* burden than mice infected with an isogenic strain lacking the plasmid [115]. Questions arise from this study regarding whether there are interactions between determinants on pX18–498 and the core genome, as well as whether specific concentrations of vancomycin in certain niches favor colonization and survival with low-level resistant mutants.

### 4.3. Fidaxomicin Resistance 

In May 2011, fidaxomicin received approval from the Food and Drug Administration (FDA) for treating CDI [58,116]. As a bactericidal antibiotic, fidaxomicin displays a lower minimum inhibitory concentration (MIC) in vitro against *C. difficile* strains, including NAP1/B1/027, in comparison to metronidazole or vancomycin [59,60].

Fidaxomicin stands out for its potential advantages in treating CDI. Unlike vancomycin, which is bacteriostatic, fidaxomicin is bactericidal [61,92]. When taken orally, both fidaxomicin and vancomycin have limited absorption from the gastrointestinal tract, leading to high fecal concentrations that surpass the MIC for *C. difficile* [58,61,92,117]. In contrast, metronidazole is almost entirely absorbed in the proximal jejunum, contributing to fecal concentrations above the MIC for *C. difficile* only when stools remain unformed [55,100]. Fidaxomicin exhibits a notably narrow spectrum of antimicrobial activity compared to vancomycin and metronidazole, resulting in a lesser impact on the normal intestinal microbiota [59].

Fidaxomicin, classified as a macrolide antibiotic, exerts its bactericidal effects by inhibiting bacterial RNA polymerase, consequently impeding transcription and subsequent protein synthesis [118]. While resistance to fidaxomicin in *C. difficile* is not widely documented, there is a reported instance of a *C. difficile* strain, isolated from a patient with rCDI, exhibiting reduced susceptibility [110,119].

Two separate studies identified associations between *C. difficile* resistance to fidaxomicin and induced mutations in the RNA polymerase subunit β (*rpoB*) during in vitro investigations (Figure 1). Specifically, in one study, the A3221G mutation in *rpoB* led to the Gln1073Arg substitution [92]. In another study, genetically engineered mutations—T3428A, T3428G and G3427C in *rpoB*—resulted in Val1143Asp, Val1143Gly and Val1143Phe substitutions, respectively [120]. Notably, the latter three mutations were observed concurrently with diminished in vivo virulence and in vitro fitness [120].

In a different investigation, mutants resistant to fidaxomicin were discovered to harbor a frameshift mutation in CD22120, a homolog of *MarR* (multiple antibiotic resistance regulator) [92]. However, the conclusive validation of the mutation’s role in fidaxomicin resistance necessitates molecular genetic confirmation.

### 4.4. Rifamycins Resistance

Rifamycins like rifaximin and rifampicin are being explored as supplementary treatments for CDI. Rifaximin has been suggested as a subsequent therapy following the initial treatment with vancomycin for recurrent CDI [8,54,70]. Rifamycins work by inhibiting bacterial DNA-dependent RNA polymerase [121]. The primary site for mutations causing resistance is the bacterial RNA polymerase *RpoB*, particularly its β-subunit [120,121]. These mutations can either interfere with direct interaction between the target and the antimicrobial molecule or alter the rifamycin-binding pocket [121].

Rifamycin resistance in *C. difficile* has been documented in various countries [122,123]. Mutations located in the rifamycin resistance-determining region (RRDR) of *rpoB*, identified in clinical *C. difficile* isolates, have been linked to rifamycin resistance, potentially causing a reduction in drug binding [120,122]. There is a suggestion that resistance to rifaximin may develop during CDI therapy, leading to clinical failure, given *C. difficile*’s mutation frequency of approximately 10^8^ to rifaximin [120,124]. This mutation frequency can give rise to high-level resistant mutants without significant impacts on in vitro or in vivo fitness.

Numerous mutations, including the frequently observed Arg505Lys, as well as Asp492Tyr, Ser507Leu, Ser488Tyr, Ser550Tyr, His502Asn, Leu584Phe, His502Tyr, Ser550Tyr, Gln489Leu and Gly510Arg, have been identified in strains resistant to rifamycins [120]. Nevertheless, most of these mutations did not impose a fitness cost on the bacteria in vitro, suggesting that additional unidentified mechanisms might contribute to rifamycin resistance in *C. difficile* [120]. 

It is essential to note that the described resistance mechanisms in CDI can have varying effects on clinical outcomes. Take vancomycin resistance, for example: an elevated MIC in vitro, classified as “resistant” based on CLSI or FDA criteria, may not necessarily result in treatment failure. This is because the oral administration of vancomycin leads to high concentrations in the gastrointestinal tract, potentially exceeding the in vitro MIC. Similar considerations apply to oral doses of fidaxomicin. The ongoing debate regarding the implications of resistance, particularly elevated MICs, and its correlation with treatment failure often centers on the concentrations of antibiotics in the gastrointestinal tract.

## 5. Conclusions

Antibiotic resistance in *C. difficile* is a global concern, marked by a rise in multidrug resistance (MDR) and the emergence of novel, often more virulent, strains worldwide. The evolution of antibiotic resistance in *C. difficile* continues as it acquires new resistance-determining mechanisms. In addition to toxigenic strains, non-toxigenic *C. difficile* strains are gaining significance as a notable reservoir of antibiotic resistance. These strains, prevalent in the natural environment, can colonize both humans and animals, thereby playing a substantial role in disseminating antibiotic resistance. In this regard, continuous surveillance of antibiotic resistance in *C. difficile* isolates from patients is crucial for comprehending the epidemiology and evolution of *C. difficile*. Moreover, public health surveillance focusing on genomics is essential for understanding and addressing the MDR in *C. difficile*, given its high diversity, mobile resistome and the continual discovery of new resistance mechanisms. Along with monitoring antibiotic resistance over time, practicing antibiotic stewardship and judicious use of antimicrobial agents with minimal impact on beneficial gut bacteria are essential strategies to address the problem. Ongoing research into the resistance mechanisms of *C. difficile*, as well as the development of new antimicrobial agents effective against *C. difficile*, is imperative. Additionally, the pursuit of alternative therapies that boost the host immune response and support gut microbiota and its associated metabolites for CDI should be considered. Ultimately, an effective vaccine would be the most effective way of preventing CDI-associated morbidity and mortality. No FDA-approved *C. difficile* vaccine currently exists; however, clinical trials and research into the development of an effective vaccine against CDI are ongoing [125,126,127].
